# A study on the association between the inferior nasal turbinate volume and the maxillary sinus mucosal lining using cone beam tomography

**DOI:** 10.1016/j.heliyon.2022.e09190

**Published:** 2022-03-26

**Authors:** Shishir Ram Shetty, Saad Wahby Al-Bayatti, Sausan Al Kawas, Natheer Hashim Al-Rawi, Vinayak Kamath, Raghavendra Shetty, Sunaina Shetty, Vijay Desai, Leena David

**Affiliations:** a College of Dental Medicine, University of Sharjah, Sharjah, United Arab Emirates; b Goa Dental College and Hospital, Goa, India; c College of Dentistry, Ajman University, Ajman, United Arab Emirates; d College of Health Sciences, University of Sharjah, Sharjah, United Arab Emirates

**Keywords:** Turbinates, Maxillary sinus, Volume, Cone-beam computed tomography, Sinusitis

## Abstract

**Objectives:**

The volume of the inferior turbinates (IT) and the lining of the maxillary sinuses are important parameters when assessing sino-nasal diseases. However, no radiographic studies have investigated the correlation between these parameters. The present study was conducted to analyze the association between IT volumes and thickness of maxillary sinus mucosal lining.

**Materials and methods:**

A retrospective evaluation of the cone-beam computed tomography (CBCT) scans of 100 adult subjects was carried out by two radiologists. The scans were assigned to two groups (Group 1 & 2) based on the radiographic evidence of maxillary sinus lining in the CBCT scans. Group 1 consisted of 50 CBCT scans of subjects with no evidence of maxillary sinus mucosal lining, whereas Group 2 consisted of 50 subjects with evidence of maxillary sinus mucosal lining. The scans in the Group 2 were further sub-classified into five categories, based on the thickness of the maxillary sinus mucosal lining. Vesalius 3D software was used to evaluate the total volume of inferior nasal turbinates by the two radiologists and a mean volume was obtained for each study subject.

**Results:**

The intraclass correlation coefficient (ICC) between the volumetric estimations performed by the two radiologist was 0.87. Analysis of the results revealed that there was no significant gender-based difference (Group 1, P = 0.67 and Group 2, P = 0.95) in the total turbinate volume in either of the study groups. The total turbinate volume did not show any significant correlation (Group 1, r = 0.24 and Group 2, r = 0.12) with the age. There was a significant increase (P = 0.001) in the total turbinate volume of the subjects in Group 2 compared to Group 1. Regression analysis revealed that the thickness of sinus lining correlated significantly (P = 0.001) with the total turbinate volume.

**Conclusion:**

An increase in the total turbinate volume has been associated with an increase in the thickness of the maxillary sinus mucosal lining. The data from this study will be useful for post-operative follow-up of inferior turbinates and maxillary sinus lining after the turbinate volume reduction procedures.

## 1 Introduction

Inferior turbinate hypertrophy (ITH) is caused by the enlargement of either bony or soft tissue components of the inferior nasal turbinates. ITH is often associated with chronic inflammatory disorders, such as rhinitis [1]. Rhinitis is associated with sneezing, itching, nasal discharge, and nasal mucosal swelling. The nasal mucosal swelling results in decreased maxillary sinus drainage and causes maxillary sinusitis [2]. Maxillary sinusitis is associated with the thickening of the maxillary sinus mucosal lining [3]. Researchers have found similarities in the inflammation associated histopathological features of the maxillary sinus mucosal lining and the hypertrophied inferior turbinate mucosa [4, 5]. Based on this information it can be hypothesized that the morphological changes of the ITH may be associated with morphological changes in the mucosal lining of the maxillary sinuses. Recent studies used Cone Beam Computed tomography (CBCT), to study the thickness of the sinus mucosa and the thickness of inferior turbinates [6, 7, 8, 9, 10]. Furthermore, studies also reported that the volumes of the inferior turbinate (IT) and the status of the maxillary sinus are important indicators for physicians to evaluate the sino-nasal diseases [11, 12]. In the past, studies have highlighted the importance of volumetric analysis of IT after turbinate reduction procedures [13].

However, to the best of our knowledge, no radiographic studies have evaluated the association or correlation of the volume of IT with the thickness of the maxillary sinus mucosal lining and dental status. Therefore, we felt that there was a need for an imaging study to determine the association between IT volumes and thickness of the maxillary sinus mucosa. In addition, there is a paucity of data in the literature pertaining to volumetric changes of ITH with respect to gender and age. Hence, our study also aimed to determine whether age and gender affect IT volume.

## 2 Material and methods

### 2.1 Study design and study population

A retrospective evaluation of 100 CBCT scans of subjects who had attended University Dental Hospital, Sharjah (UDHS) clinics for various dental treatments from January 2017 to December 2020 was carried out. The study was approved by the human subjects ethics board of the University of Sharjah (Reference number: REC-21-01-10-01) and was conducted in accordance with the Helsinki Declaration of 1975, as revised in 2013 [14]. CBCT scans of male and female study subjects aged between 18 to 75 years were included in the study. Scans with artifacts and incomplete anatomical coverage of the area of interest were excluded. CBCT scans of subjects with history of midfacial trauma, maxillary sinus-related surgical procedures and syndromes affecting the orofacial skeleton were excluded from the study.

Sample size estimation (n = 100) was done using statistical Software G∗Power 3.1. Based on the observation made from previous literature by El-Anwar et al [7], considering effect size of 0.24, 80% power, and α error of 5%, a sample size of 48 was calculated which was rounded off to 50 subjects per group. The CBCT scans were categorized into two groups.

Groups 1 50 subjects with no radiographic evidence of maxillary sinus lining in the CBCT scans.

Group 2 50 subjects with radiographic evidence of maxillary sinus lining in the CBCT scans.

The ethnicity of the study subjects was classified as Arab and Non-Arab based on the classification by Abuelezam et al [15]. The past medical history of the study subjects was evaluated for evidence of chronic rhinosinustis using hospital's electronic health record system. The subjects were classified as positive or negative for the history of chronic rhinosinusitis based on the criteria stated by Ah-See et al [16].

**Figure 1:**
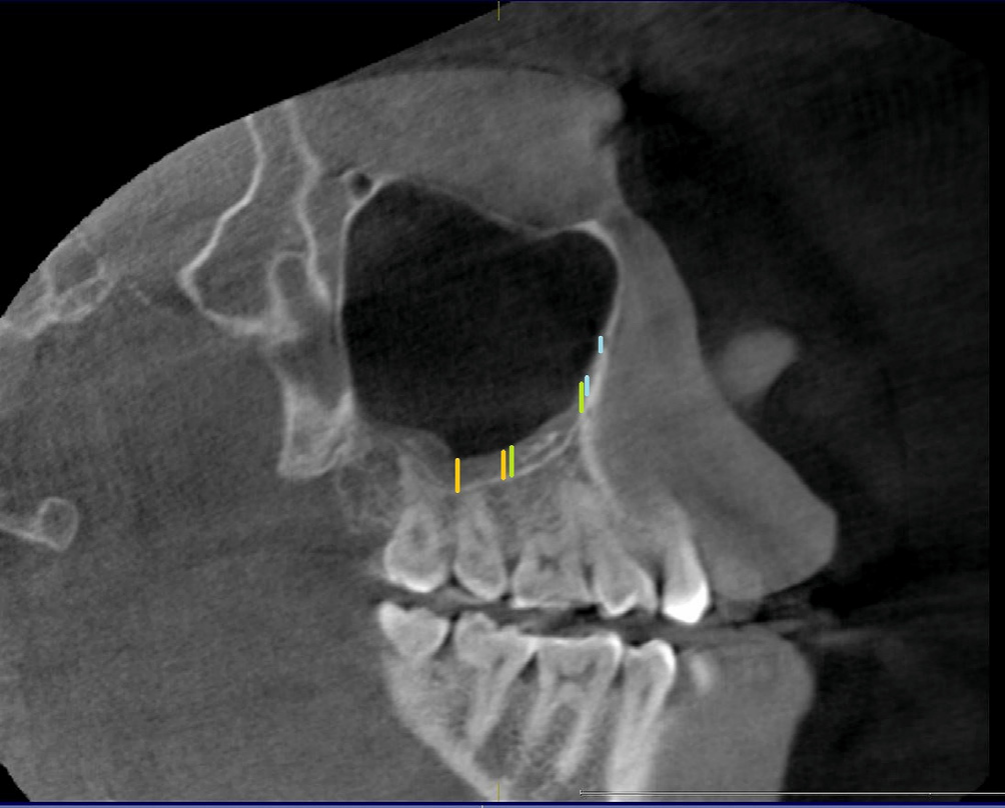
Sagittal CBCT section showing the six sites at which the mucosal thickening was measured in dentate study subjects. The mesial and distal sides of the second.

**Figure 2:**
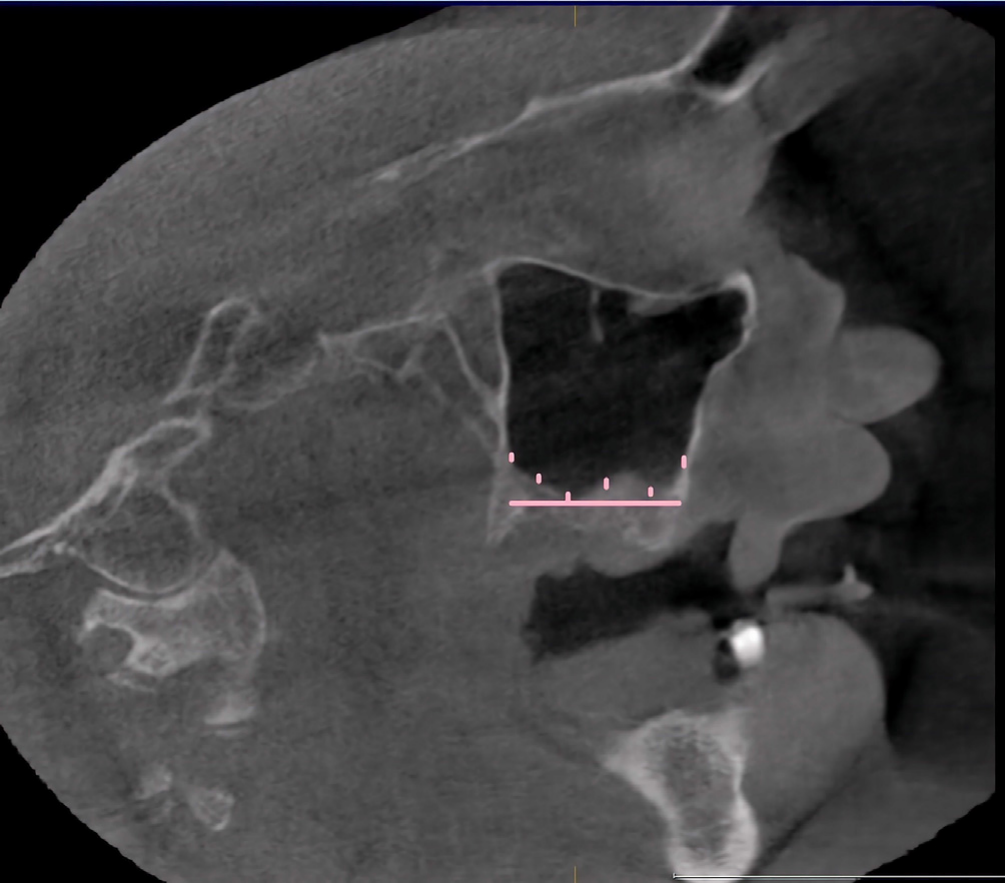
Sagittal CBCT section showing the sites at which the mucosal thickness was measured in edentulous study subject. Six equidistant sites (vertical pink lines) on an.

For the subjects in Group 2, the mucosal lining measurements were carried out using the method adopted by Shetty et al [17] and Sheikhi et al [8]. According to the criteria the mucosal thickening was evaluated at six locations on the floor of each maxillary sinus, in the sagittal CBCT section (Figure 1). The measurements were made at mesial and distal sides of the second premolar, first and second molar. For edentulous study subjects the measurements were made at six equidistant sites on an imaginary line connecting the anterior and posterior most points on the floor of the sinus in sagittal section (Figure 2).

The subjects were categorized (type 1 to 5) based on the maximal thickness of the mucosal lining measured at these six sites in the right and left maxillary sinuses. To avoid age and gender-based variations, subjects in Group 1 were matched according to age and gender with subjects in Group 2.

The dental status of the maxillary arch from first premolar to second molar was examined in the CBCT scans of the study subjects. The scans were classified as dentulous, partially edentulous, completely edentulous based on the findings in the arch.

### 2.2 Image acquisition and evaluation

CBCT scans were obtained using Galileos CBCT unit (Sirona Dental Systems, Bensheim Germany). The image acquisition parameters were as follows; Field of View (15 cms × 24 cms), voxel size (0.25 mm), 85 kVp and 7 mA. The image was viewed using Sidexis software on a 1920 × 1080 pixel, 23-inch DELL monitor screen.

Two dental radiologists with 10 years experience analyzed the CBCT images. A third examiner with equivalent expertise was consulted in case of a disagreement between the two primary examiners.

### 2.3 Total turbinate volume detection

**Figure 3:**
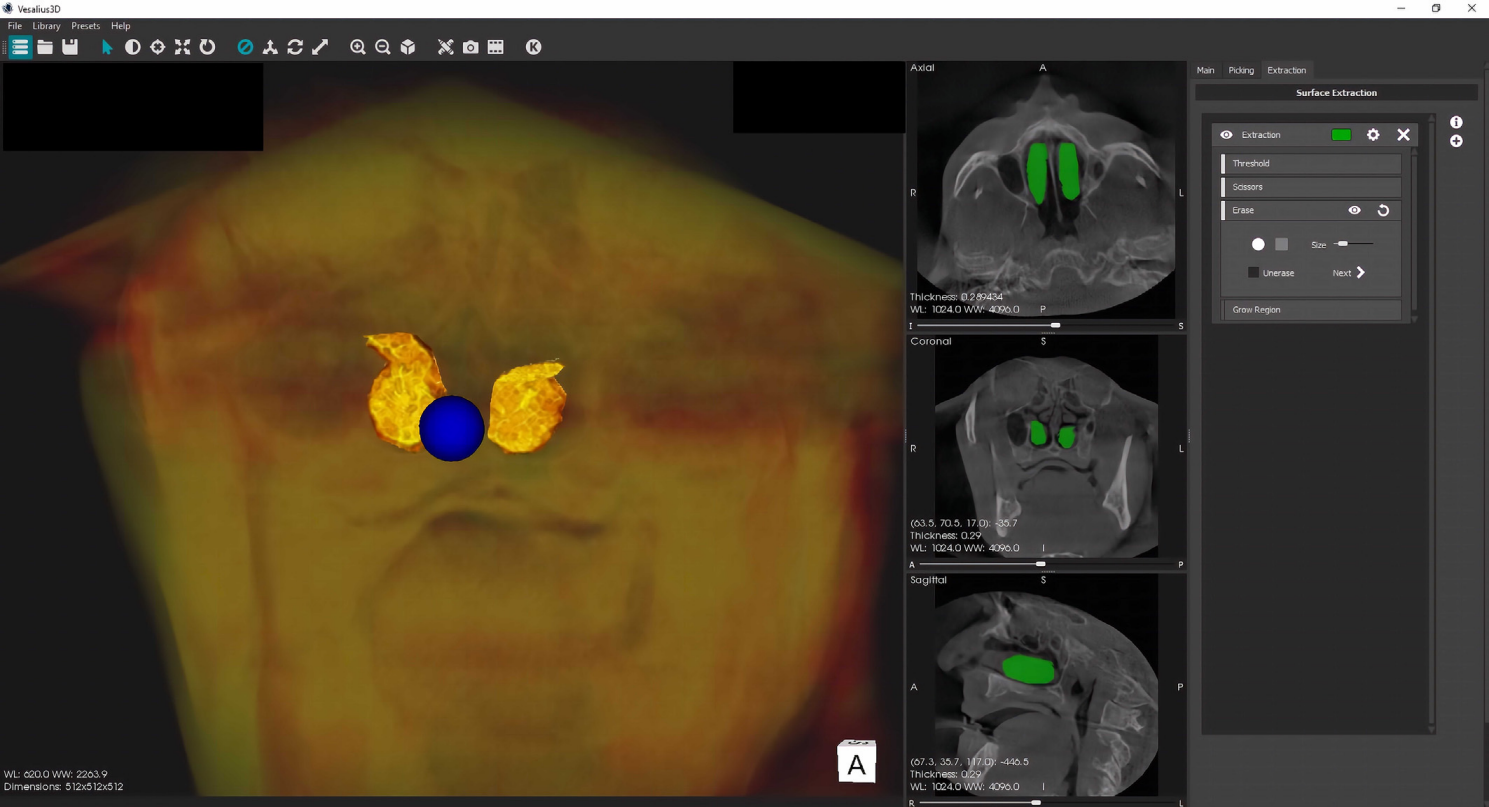
Final phase of inferior nasal turbinate segmentation procedure using eraser tool.

**Figure 4:**
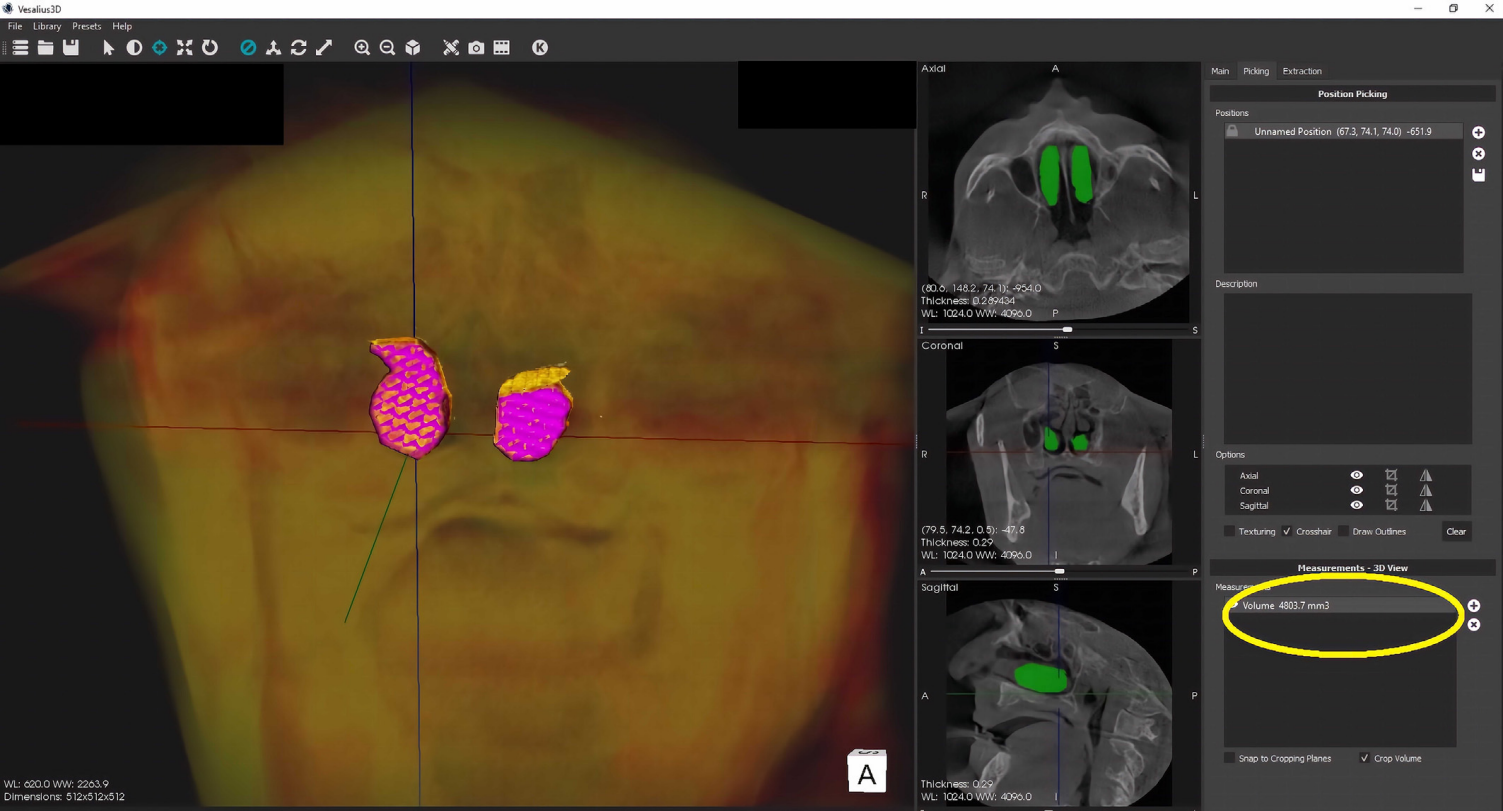
Volume determination of the segment inferior nasal turbinates. The yellow circle shows the volume after segmentation.

The scans of the study subjects were exported and saved in the Digital Imaging and Communications in Medicine (DICOM) format. The scans were then imported into the Vesalius 3D software (PS-Medtech, Amsterdam, Netherlands). The CBCT scans were then visualized using contrast settings. The inferior nasal turbinates were then segmented using surface extraction tools such as scissors and erase functionality (Figure 3). For standardization, the segmentation process included the entire turbinate till the contact surface on the lateral nasal wall. The smooth surface functionality in the extraction settings was used to reduce surface irregularities of the turbinates. After completing segmentation, picking option on the software toolbar was selected. In the next step, the total turbinate volume of the inferior nasal turbinates was measured using the extract volume functionality (Figure 4). The volume determination was carried out by the two examiners separately to assess reliability. A mean total turbinate volume was determined using the volumetric data from the two examiners.

The study variables: age of the subjects, gender of the subjects, total turbinate volume measurements, and the type of maxillary sinus mucosal lining were statistically analyzed using IBM SPSS statistics (Version 22, Armonk. NY: IBM Corp).

The raw data is available at figshare; doi:10.6084/m9.figshare.16881970.

## 3 Results

In this study, two oral radiologists evaluated radiographic evidence for sinus mucosal lining in 100 CBCT scans. The intraclass correlation coefficient (ICC) between the volumetric estimations performed by the two radiologist was 0.87. The electronic medical records of these 100 study subjects were evaluated and analyzed. When the ethnicity of the subjects was compared in the two study groups (Group 1 and 2), no significant (p = 0.83) difference was observed between the Arab and Non-Arab subjects (Table 1). There were a significantly higher (p = 0.01) number of study subjects with a positive history of rhinosinusitis in group 2 compared to Group 1(Table 2). This finding suggested that there are higher chances of finding radiographic evidence of maxillary sinus mucosal thickening in the individuals with a history of rhinosinusitis. Among the 100 CBCT scans, 65 scans belonged to males and 35 belonged to females. There was no significant difference in the gender-distribution between the two study groups (p = 0.83) (Table 3). Evaluation of the dental records revealed no significant difference in the dental status (dentulous/partially edentulous/completely edentulous) between the study groups (p = 0.58) (Table 4). When the total turbinate volume of the male and female subjects in the two study groups was compared, no statistically significant difference was observed (Group 1, p = 0.67 and Group 2, p = 0.95) (Table 5). These findings suggest that the gender of the study subjects had no significant effect on the total turbinate volume. When the total turbinate volume of the dentulous, partially edentulous and completely edentulous subjects in the two study groups were compared, no significant difference was observed (Group 1, p = 0.61 and Group 2, p = 0.55) (Table 6). Though the difference was not significant, the mean total turbinate volume of dentulous subjects was higher than those of partially and completely edentulous subjects.

**Table 1:** Comparison of the ethnicity of the study subjects in Group 1 and Group 2.

		Group		Total	Chi Square Test	
		1	2		Chi Square value	p-value
Ethnicity	Arab	27	29	56	0.04	0.83(NS)
		54.0%	58.0%	56.0%		
	Non-Arab	23	21	44		
		46.0%	42.0%	44.0%		

∗p < 0.05 statistically significant. p > 0.05 non significant, NS.

**Table 2:** Comparison of the history of rhinosinusitis in the study Group 1 and Group 2.

		Group		Total	Chi Square Test	
		1	2		Chi Square value	p-value
History of rhinosinusitis	Positive	8	20	28	6.63	0.01∗
		54.0%	58.0%	28.0%		
	Negative	42	30	72		
		46.0%	42.0%	72.0%		

∗p < 0.05 statistically significant. p > 0.05 non significant, NS.

**Table 3:** Gender wise comparison of the subjects in the study groups.

		Group		Total	Chi Square Test	
		1	2		Chi Square value	p-value
Gender	Male	32	33	65	0.04	0.83(NS)
		64.0%	66.0%	65.0%		
	Female	18	17	35		
		36.0%	34.0%	35.0%		

∗p < 0.05 statistically significant. p > 0.05 non-significant, NS.

**Table 4:** Comparison of dental status between study groups.

Dental status	Group		Total	Fisher's Exact Test
	1	2		p-value
Completely edentulous	1	3	4	0.58(NS)
	2.0%	6.0%	4.0%	
Partially edentulous	27	24	51	
	55.1%	48.0%	51.5%	
Dentulous	21	23	44	
	42.9%	46.0%	44.4%	
Total	49	50	99	
	100.0%	100.0%	100.0%	

∗p < 0.05 Statistically Significant. p > 0.05 Non Significant,NS.

**Table 5:** Comparison of total turbinate volume between the male and female subjects of the study groups.

Group	Gender	N	Mean	SD	Mean Difference	95% Confidence Interval of the Difference		t	df	p-value
						Lower	Upper			
Group 1	Male	32	3242.94	312.48	40.29	-147.31	227.89	0.43	48	0.67(NS)
	Female	18	3202.65	324.21						
Group 2	Male	33	4446.95	652.81	13.25	-397.34	423.83	0.07	48	0.95(NS)
	Female	17	4433.70	742.49						

Independent sample t test.

∗p < 0.05 statistically significant. p > 0.05 non-significant, NS.

**Table 6:** - Comparison of the turbinate volume in relation to the dental status.

Group	Dental status	N	Mean Turbinate volume	SD	Mean Difference	95% Confidence Interval of the Difference		T	df	p-value
						Lower	Upper			
1	Completely + Partially edentulous	28	3195.54	337.62	46.54	-137.29	230.36	0.51	47	0.61(NS)
	Dentulous	21	3242.07	299.95						
2	Completely + Partially edentulous	27	4379.03	655.45	117.43	-271.34	506.20	0.61	48	0.55(NS)
	Dentulous	23	4496.46	702.6						

Independent sample t test.

∗p < 0.05 Statistically Significant. p > 0.05 Non Significant, NS.

Further, when the mean age of the subjects in the study groups was compared, no significant difference (p = 0.7) was observed (Table 7). When the age of the subjects was correlated with the total turbinate volume, there was no significant correlation was found in either study group (Table 8). This finding suggested neither the age of the study subjects nor their mucosal type of maxillary sinus affected the total turbinate volume.

**Table 7:** Comparison of age between the subjects in the study groups.

Group	N	Mean	SD	Mean Difference	95% Confidence Interval of the Difference		t	df	p-value
					Lower	Upper			
Group 1	50	51.84	14.43	13.42	8.02	18.82	4.93	98	0.7(NS)
Group 2	50	52.42	12.73						

Independent sample t test.

∗p < 0.05 statistically significant. p > 0.05 non-significant, NS.

**Table 8:** Correlation between total turbinate volume and age of the subjects in study groups.

Group		Age
1	r	0.28
	p-value	0.05(NS)
2	r	0.12
	p-value	0.42(NS)

Pearson's Correlation Test.

∗p < 0.05 statistically significant. p > 0.05 non-significant, NS.

When the total turbinate volume of the subjects in the two study groups was compared, a statistically significant (p = 0.001) association was observed (Table 9). Group 2 had a significantly higher total turbinate volume compared to Group 1, suggesting that subjects with radiographically evident maxillary sinus lining had higher total turbinate volume.

**Table 9:** Comparison of total inferior turbinate volume between the study groups.

Group	N	Mean	SD	Mean Difference	95% Confidence Interval of the Difference		t	df	p-value
					Lower	Upper			
Group 1	50	3228.44	314.04	-1214.01	-1423.46	-1004.56	-11.50	98	0.001∗
Group 2	50	4442.45	677.03						

Independent sample t test.

∗p < 0.05 statistically significant. p > 0.05 non-significant, NS.

In study Group 2, type 3 sinus mucosal lining had the most common occurrence among the subjects, whereas type 5 sinus mucosal lining was the least (Table 10). In Group 2, there was a statistically significant difference in the total turbinate volume was associated with different types of maxillary sinus mucosal lining when compared using ANOVA. Type 5 mucosal lining was associated with significantly higher total turbinate volume, while Type 1 was associated with the lowest total turbinate volume (Table 11). The pairwise comparison of the total turbinate volume associated with each type of mucosal lining confirmed a significant difference in the total turbinate volume among all the types of sinus mucosal lining (Table 12).

**Table 10:** Distribution of study subjects in group 2 according to type of sinus lining.

Type of mucosal lining	Number of subjects	Percentage of subjects
Type 1	8	16 %
Type 2	10	20%
Type 3	15	30%
Type 4	11	22%
Type 5	6	12 %

**Table 11:** Comparison of total inferior turbinate volume according to type of sinus lining.

Type of sinus lining	N	Mean	SD	Min	Max	ANOVA	
						F	p-value
Type 1	8	3495.95	201.99	3100.31	3688.32	114.45	0.001∗
Type 2	10	4056.28	151.16	3785.66	4288.56		
Type 3	15	4364.18	433.32	3111.03	4667.60		
Type 4	11	5116.29	100.07	4932.11	5288.45		
Type 5	6	5308.33	446.47	4577.74	5722.01		

∗p < 0.05 statistically significant. p > 0.05 non-significant, NS.

**Table 12:** Pairwise comparison of total inferior turbinate volume according to type of sinus lining.

(I) Type of sinus lining	(J) Type of sinus lining	Mean Difference (I-J)	Std. Error	p-value	95% Confidence Interval	
					Lower Bound	Upper Bound
Type 1	Type 2	-560.33	147.18	0.003∗	-988.51	-132.14
	Type 3	-868.23	135.84	0.001∗	-1263.43	-473.03
	Type 4	-1620.34	144.17	0.001∗	-2039.78	-1200.89
	Type 5	-1812.38	167.57	0.001∗	-2299.89	-1324.87
Type 2	Type 3	-307.90	126.67	0.001∗	-676.42	60.62
	Type 4	-1060.01	135.57	0.001∗	-1454.42	-665.59
	Type 5	-1252.05	160.22	0.001∗	-1718.20	-785.91
Type 3	Type 4	-752.11	123.17	0.001∗	-1110.44	-393.78
	Type 5	-944.15	149.88	0.001∗	-1380.20	-508.11
Type 4	Type 5	-192.05	157.47	0.83(NS)	-650.18	266.09

Tukey Post Hoc Test.

∗p < 0.05 statistically significant. p > 0.05 non-significant, NS.

Regression analysis revealed that the total turbinate volume had a significant association (P = 0.001) with the type of maxillary sinus lining, whereas the age (P = 0.42) and the gender (P = 0.26) of the study subjects did not show significant association (Table 13).

**Table 13:** Linear Regression to predict total inferior turbinate volume based on study variables.

	Unstandardized Coefficients		Standardized Coefficients	t	p-value	95.0% Confidence Interval for B	
	B	Std. Error	Beta			Lower Bound	Upper Bound
Constant	3067.18	147.39		20.81	0.001∗	2774.61	3359.76
Gender	-74.14	65.85	-0.04	-1.13	0.26(NS)	-204.86	56.57
Age	4.65	2.31	0.09	2.01	0.42 (NS)	0.06	9.24
Type of sinus lining	448.03	20.29	0.96	22.08	0.001∗	407.76	488.31

F(3,96) = 186.00, p < 0.001, R^2^ = 0.85.

## 4 Discussion

The inferior turbinate is the largest of the three turbinates arising from the lateral wall of the nose. It is composed of bony component and a soft tissue component with a thick mucous membrane containing a cavernous plexus. The inferior turbinate contributes in thermoregulation, humidification and filtration of air [18].

ITH is usually caused by infections, allergies, exposure to irritants, smoking, vasomotor rhinitis deviated septum and chronic infection in the sinuses [19]. Some case reports have highlighted the presence of thickened maxillary sinus mucosal lining in cases with hypertrophy or pneumatization of inferior turbinates [20, 21]. Although the correlation between sinus mucosal thickening and IT hypertrophy is physiologic, there was no radiographic study highlighting the association between the volume of the IT and thickening of the maxillary sinus mucosal lining and dental status. In our study we have evaluated the IT volumes and thickening of the sinus mucosal with relation to the age, gender, ethnicity and dental status of the study subjects. We have also analyzed the association between IT volumes (total turbinate volume) and thickness of maxillary sinus mucosal lining. The data obtained from our study will be useful for the post-operative radiographic evaluation of the inferior turbinates and sino-nasal structures following turbinate volume reduction. The data from turbinate volume analysis data could be useful in the management of nasal septal deviation [22].

In the present study there was no significant difference between the occurrence of thickening in the sinus mucosal lining between Arab and non-Arab study subjects. Some researchers have reported of differences in the occurrence of sino-nasal variations and rhinosinusitis among different ethnicities [23]. The probable reason for the lack of significant difference in this parameter among the groups in our study could be due to the heterogenous ethnicity in the Non-Arab subjects, owing to a large expat population in the region of study.

Vesalius 3D software was used for the purpose of segmentation and volume determination of the ITH in our study. Contemporary studies have proven that Vesalius 3D provides accurate volumetric analysis of the anatomic structures [24]. A recent study reported that the error margin for measurement on Vesalius 3D was less than 0.5% [25].

Recently, three dimensional volumetric analysis of the anatomical structures in the CBCT images have been carried out using specialized softwares [26, 27, 28, 29, 30, 31]. Most of the volumetric analysis software's are semi-automated and few of them are fully automated [32]. In the present study, the segmentation procedure was carried out manually using tools like scissors and erasers available in the Vesalius 3D software. The Vesalius 3D volumetric assessment was found to be reliable in a recently conducted study [33].

In our study, we did not find a statistically significant change in total turbinate volumes when the subjects are compared by age and gender. A recent study conducted using CT also reported that there were no significant age-related linear dimensional changes in ITH [34]. Similarly, no gender-related variation was observed in a study conducted by de Bonnecaze et al while evaluating three-dimensional polymorphism of the inferior turbinates [35].

In our study, the total turbinate volume was significantly higher in the Group 2 with subjects having radiographically visible sinus lining thickness. To the best of our knowledge, no previous studies have evaluated the turbinate volume with the thickness of lining in the maxillary sinus. However, a study that measured the thickness of nasal turbinates in sinus volumes found that the sinus volumes decreased significantly with increasing turbinate thickness [36]. It is stated that the increase in turbinate size can lead to nasal obstruction and recurrent sinusitis [37]. This could be a possible reason for the increased volumes of the inferior turbinates in subjects with increased thickness of maxillary sinus mucosal lining.

In the study, we found that type 2 and type 3 maxillary sinus mucosal lining were most common in Group 2 whereas type 5 was least common. A similar pattern of distribution of the Type 5 sinus mucosal lining was observed in the study by Sheikhi et al 2014 [8].

Our study results indicate a significant increase in the total turbinate volume as the sinus mucosal thickness increased. This could be explained based on the overall sino-nasal inflammatory process. Maxillary sinus mucosal thickening is an inflammatory reaction characterized by hyperplasia of the mucous lining. Infections, allergies, and chronic rhinosinusitis are commonly associated with maxillary sinus mucosal thickening [38]. Chronic rhinosinusitis is associated with swelling of the nasal mucous membrane and inferior turbinate hypertrophy [39]. Another reason could be similarities in histopathological inflammatory changes occurring in the ITH and sinus mucosal lining. Clinico-histopathological studies have revealed similarities in the inflammatory component of the ITH and thickened maxillary sinus mucosal lining [5, 40].

One of the major highlights of this study is the application of semi-automated software (Vesalius 3D) for measuring volumes of ITH. In a recent study, it was identified that semi-automated volumetric measurements are more replicable and accurate than linear measurements for measuring anatomical structures, and the difference was very obvious when anatomical structures had an oblique orientation [41]. This fact justifies the application and accuracy of the volumetric measurements in the present study.

Though volumetric analysis provides more accurate dimensions, a longer time is required for the segmentation and volumetric analysis of the anatomical structures. In the present study, each semi-automated segmentation procedure along with volumetric estimation required approximately 30 min. Researchers are working on artificial intelligence-based fully automated segmentation of IT. We believe that the volumetric data from our study can be very useful for further research work using fully automated segmentation.

A recent study has revealed that there is an association between dental status and mucosal thickening [42]. However, there are no studies that have examined the association between total turbinate volume and dental status. Results of the present study revealed that there was no significant difference in the total turbinate volume and the dental status of the study subjects.

One of the limitations of our study is the inability to distinctly evaluate the volumes of the bony and soft tissue components of the IT. ITH can be primary and secondary in nature. The former is associated with the soft tissue component, while the latter is associated bony component of the turbinate and caused due to nasal septal deviation [43]. Further research can be carried out for determining the volumes of bony and soft tissue components of the ITH distinctly. This would aid in determining the medical or surgical line for the reduction of turbinate volume.

## 5 Conclusion

The results of this study demonstrated that an increase in the total turbinate volume is associated with an increase in the thickness of the maxillary sinus mucosal lining. Findings of this study will be useful to the physicians during the post-operative follow-up and evaluation of the inferior turbinates and sino-nasal structures after volume reduction of the IT.

## 6 Ethical approval

All methods were carried out in accordance with relevant guidelines and regulations. All experimental protocols were approved by a named institutional ethical committee obtained Ref. no. REC-21-01-10-01 (Institutional review board, University of Sharjah).

## 7 Informed consent

Informed written consent was obtained from all subjects involved in the study.

## Declarations

### Author contribution statement

Shishir Ram Shetty: Conceived and designed the experiments; Performed the experiments; Analyzed and interpreted the data; Contributed reagents, materials, analysis tools or data; Wrote the paper.

Saad Wahby Al-Bayatti, Sausan Al Kawas, Natheer Hashim Al-Rawi, Raghavendra Shetty, Sunaina Shetty, Leena David: Contributed reagents, materials, analysis tools or data; Wrote the paper.

Vinayak Kamath: Analyzed and interpreted the data.

Vijay Desai: Analyzed and interpreted the data; Wrote the paper.

### Funding statement

This work was supported by 10.13039/100016714University of Sharjah (V.C.R.G./R.438/2020 (Project number 2101100146)).

### Data availability statement

Data associated with this study has been deposited at figshare; doi:10.6084/m9.figshare.15086688.

### Declaration of interests statement

The authors declare no conflict of interest.

### Additional information

No additional information is available for this paper.
