# Corrigendum to “A study on the association between the inferior nasal turbinate volume and the maxillary sinus mucosal lining using cone beam tomography” [Heliyon Volume 8, Issue 3, MARCH 2022, Article e09190]

**DOI:** 10.1016/j.heliyon.2024.e34459

**Published:** 2024-07-14

**Authors:** Shishir Ram Shetty, Saad Wahby Al-Bayatti, Sausan Al Kawas, Natheer Hashim Al-Rawi, Vinayak Kamath, Raghavendra Shetty, Sunaina Shetty, Vijay Desai, Leena David

**Affiliations:** a College of Dental Medicine, University of Sharjah, Sharjah, United Arab Emirates; b Goa Dental College and Hospital, Goa, India; c College of Dentistry, Ajman University, Ajman, United Arab Emirates; d College of Health Sciences, University of Sharjah, Sharjah, United Arab Emirates

In the original published version of this article, we recently received the comment below from a reader.

Dear editor.

I read with great interest the study by Shetty et al. (2022) 1 published in your journal. It was very interesting except that its main statistical analyses (in Tables 9 and 13) and hence its conclusions were incorrect: It is important to note that the authors had matched the two groups of the study in terms of the subjects' age and sex. Such a matching removed the assumption of ‘independent observation’. Independent-samples tests assume the independence of observations. Thus, a matched design calls for paired tests for the comparisons between the groups mentioned in Tale 9 and mixed-model linear regressions for Tale 13. Nevertheless, the authors used an independent samples test and simple linear regression, which are incorrect for such a methodology. Therefore, I would love it if the authors corrected their analyses and conclusions accordingly.

So, we contacted the paper's handling editors, who evaluated and made a decision, and requested their feedback. They said that they required the opinion of a professional biostatistician for additional analysis. We called one to acquire confirmation, and the results of their analysis are listed below.

Conditional regression, and more generally statistical analysis, is a well-known technique to eliminate possible confounders; see a review by Chia-Ling Kuo,Chia-Ling Kuo, Yinghui Duan, Yinghui Duan, James Grady (2018) “Unconditional or Conditional Logistic Regression Model for Age-Matched Case–Control Data?“, Frontiers in Public Health. It was mathematically shown that the paired *t*-test and regression slope coefficients do not change compared to the traditional analysis. This means that the results of the study are valid although the authors should make a comment on the rightfulness of their approach and provide a relevant reference.

Based on the above comments, we requested the authors to draft an update to their rationale as suggested and submit it as a correction paper in Heliyon.

**Author Correction**.

Dear Editor.

The response to the comment raised by the reader regarding the statistical analysis of our study published in Heliyon is as follows [1]. In a review by Chia-Ling Kuo et al. it was mathematically shown that the paired *t*-test and regression slope coefficients do not change compared to the traditional analysis [2].

Matched analysis may be performed only for genuinely matched data. When matching is based solely on a few demographic variables, such as age and gender, the resulting data is typically loosely matched. For loosely matched data, matched methods are not necessary, and the data can be effectively analyzed using unmatched methods [2,3].

## Declaration of competing interest

The authors declare no conflicts of interest.
